# Family Affluence and Lifestyle Behaviors as Determinants of Fat Mass Index in University Students: A Sex-Specific Structural Equation Modeling Approach

**DOI:** 10.3390/nu18050730

**Published:** 2026-02-25

**Authors:** Jarosław Domaradzki

**Affiliations:** Department of Biological Principles of Physical Activity, Wroclaw University of Health and Sport Sciences, 51-612 Wrocław, Poland; jaroslaw.domaradzki@awf.wroc.pl

**Keywords:** dietary behaviors, physical activity, sedentary time, family affluence, structural-equation modeling, sex differences, university students

## Abstract

**Background/Objectives**: Family affluence is commonly considered an important contextual determinant of adiposity; however, its relative contribution compared with current lifestyle behaviors during early adulthood remains unclear. This study examined direct associations between family affluence, specific lifestyle indicators (physical activity, sedentary time, and dietary indices), and fat mass index (FMI) in university students. **Methods**: This cross-sectional study included 418 students (199 males, 219 females). Family affluence (FAS), physical activity (PA), sedentary time (SITT), and dietary behaviors (positive and negative dietary indices) were assessed using standardized questionnaires. To evaluate how family affluence and behavioral variables relate to fat mass index (FMI), a structural equation modeling approach was implemented. Sex-specific models were estimated for males and females independently. **Results**: In both sexes, physical activity was inversely associated with fat mass index (FMI) and represented the strongest protective factor (males: β = −0.36, 95% CI: −0.46 to −0.26; females: β = −0.35, 95% CI: −0.45 to −0.25; both *p* < 0.001). The negative dietary index showed a significant positive association with FMI in males (β = 0.34, 95% CI: 0.21 to 0.48; *p* < 0.001) and females (β = 0.26, 95% CI: 0.10 to 0.42; *p* = 0.001). Sedentary time was positively associated with FMI only in females (β = 0.15, 95% CI: 0.02 to 0.28; *p* = 0.022). No statistically significant direct effect of family affluence on FMI was observed in either males or females. The SEM models explained 30% of FMI variance in males and 37% in females. **Conclusions:** In this cross-sectional university sample, proximal lifestyle indicators showed stronger direct associations with FMI than family affluence. These findings suggest that interventions targeting physical activity and unhealthy dietary behaviors may be particularly relevant for adiposity prevention in early adulthood, although longitudinal research is required to clarify causal pathways.

## 1. Introduction

Body composition trajectories are often shaped and reinforced during early adulthood, with long-term implications for later health status. During this period, lifestyle behaviors become increasingly autonomous, while earlier familial and socioeconomic influences may continue to exert long-lasting effects on adiposity. Socioeconomic status (SES), frequently operationalized through family affluence indicators, has been repeatedly associated with body composition outcomes, including body fat percentage, fat mass, and body mass index (BMI), although the direction and magnitude of these associations appear context-dependent and population-specific [1,2]. University students constitute a particularly relevant group in this regard, as they transition from family-structured environments to independent living while still reflecting earlier socioeconomic exposures [3].

Family affluence has been consistently linked to health-related behaviors established during early adulthood and carried into early adulthood. Evidence from large population-based studies indicates that higher family affluence is associated with greater participation in physical activity, healthier dietary habits, and lower prevalence of health-risk behaviors [4]. Path-analytical and mediation-based studies in early adulthood further demonstrate that SES exerts indirect effects on fat mass through family and lifestyle factors, including parental behaviors, physical activity, and shared dietary practices [5]. Similar associations have been reported in university populations, where sociodemographic background and regional origin remain linked to body composition beyond individual behaviors [1,2].

Current lifestyle behaviors represent proximal determinants of adiposity during early adulthood. Numerous studies in university students and young adults report inverse associations between physical activity levels and fat mass or body fat percentage, alongside positive associations with sedentary time [6].

Dietary patterns and eating behaviors also show robust links with adiposity, with unhealthy or snack-based dietary patterns, emotional eating, and irregular meal patterns associated with higher fat mass [7], whereas healthier dietary profiles predict more favorable body composition outcomes [8]. Longitudinal evidence summarized in systematic reviews suggests that eating patterns adopted during early adulthood contribute to later adiposity development [9].

Physical activity and dietary behaviors frequently co-occur, yet empirical findings regarding their interdependence remain inconsistent. Several investigations have reported minimal or non-significant relationships between physical activity and dietary habits, pointing toward largely distinct behavioral mechanisms [6,8]. Others report indirect or mediated relationships, particularly in intervention or pediatric samples, where physical activity may influence adiposity partly through changes in eating regulation or dietary adherence [10]. These findings suggest that physical activity and dietary behaviors may form partially overlapping but largely independent pathways in relation to body composition, particularly when examined across sexes.

Beyond physical activity and overall diet quality, daily behavioral structure in early adulthood also includes sleeping routines, meal timing, hydration patterns, and stimulant beverage consumption. Irregular sleep schedules, late-night eating, breakfast skipping, and high intake of caffeinated or energy-dense beverages have been associated with adverse adiposity indicators in young adults. Importantly, these associations frequently demonstrate sex-specific patterns, suggesting differential behavioral regulation and metabolic responses between males and females. Evidence from sex-stratified analyses indicates that sleeping and nutritional habits may interact in shaping somatic health outcomes during early adulthood [11]. Such findings further support the importance of sex-specific analytical approaches when examining lifestyle determinants of fat mass.

Most studies examining SES, lifestyle behaviors, and adiposity rely on multivariable regression models that estimate independent associations while adjusting for covariates [3,7]. Mediation and moderation analyses have been applied primarily in pediatric, clinical, or intervention contexts, often using simple regression-based approaches [5,10]. Path analysis and structural equation modeling (SEM) have been used less frequently in young adult populations, despite their suitability for simultaneously estimating direct pathways among correlated predictors [12].

The above literature indicates that previous research has largely focused either on socioeconomic determinants of adiposity or on lifestyle behaviors examined in isolation. Studies explicitly modeling multidimensional relationships among family affluence, multiple lifestyle behaviors, and adiposity within a single analytical framework are limited, particularly among physically active young adults. A gap remains in the literature regarding the simultaneous assessment of family affluence and ongoing lifestyle behaviors as determinants of FMI, particularly with respect to their relative magnitude within a structural equation modeling framework applied to university students.

To our knowledge, limited evidence is available from sex-specific structural equation modeling frameworks that simultaneously integrate family affluence with multiple concurrent lifestyle indicators in university students while using fat mass index (FMI) as the primary outcome.

Importantly, few studies have directly compared the relative hierarchy of distal socioeconomic factors and proximal lifestyle behaviors within a single multivariable SEM framework. Thus, beyond estimating independent effects, the present study seeks to determine the comparative strength and ordering of predictors of FMI when modeled simultaneously. This approach allows for a clearer evaluation of whether family affluence retains explanatory relevance once current lifestyle indicators (physical activity, sedentary time, and dietary indices)are considered.

FMI was selected as the adiposity marker because, unlike BMI, it isolates fat mass relative to height and is therefore less confounded by lean body mass, which is particularly relevant in physically active young adults. Compared with percentage body fat alone, FMI provides a height-adjusted index of adiposity that facilitates more precise modeling of behavioral determinants.

Accordingly, a structural equation modeling approach was employed to analyze the direct links between family affluence, specific lifestyle indicators (total physical activity, sedentary time, and positive and negative dietary indices), and fat mass index in a university student population. The analysis focused on the following objectives: (1) to assess associations between family affluence and physical activity and dietary behaviors; (2) examine interrelations among lifestyle behaviors to evaluate potential indirect pathways; (3) determine the direction and relative strength of direct effects of family affluence, physical activity, sedentary behavior, and dietary factors on FMI within a multivariable path model.

## 2. Materials and Methods

Data used in this study originate from a comprehensive student survey conducted in 2022–2023 and previously analyzed for different thematic purposes. The current work combines two independent cohorts and reorients the focus toward modeling the direct effects of family affluence and concurrent lifestyle factors on fat mass index. Because inclusion required complete data on all variables relevant to the present structural framework, the final sample size differs slightly from that reported in earlier articles based on the same project.

### 2.1. Study Design

This analysis is based on student data collected at Wroclaw University of Health and Sport Sciences across the 2022–2023 academic cycle. The final sample combined two cohorts enrolled in consecutive years, both of which followed an identical recruitment scheme and standardized assessment procedures. For the purposes of the present study, data from both cohorts were combined.

Before merging the two datasets, between-cohort consistency was formally assessed. Comparability of the cohorts with respect to age, anthropometric characteristics, body composition, functional measurements and questionnaires was examined using appropriate between-group statistical tests. To examine the structural alignment of variable patterns across cohorts, a Procrustes procedure was implemented. As no statistically meaningful differences were identified, data from both cohorts were pooled for the present analyses. Because the cohorts were subsequently pooled and not treated as separate analytical groups in the structural modeling stage, formal multi-group SEM invariance testing (configural, metric, or scalar) was not performed. Instead, cohort equivalence was established at the descriptive and multivariate structural level prior to data integration. No statistically significant differences were observed between cohorts in age, sex distribution, BMI, physical activity, sedentary time, dietary indices, or FAS (all *p* > 0.05). Multivariate Procrustes analysis confirmed structural similarity of variable configurations across cohorts, supporting data pooling. Detailed methodological procedures and full results are available in a previous report [13].

### 2.2. Ethics

Ethical approval for the study was granted by the Senate Research Ethics Committee of the Wroclaw University of Health and Sport Sciences (approval no. 13/2022). Before enrollment, participants were informed about the study procedures and submitted electronic informed consent.

### 2.3. Sample Size

Sample size planning was guided by standard criteria for exploratory multivariate procedures, specifically the assumption of a minimum ten-to-one ratio between observations and predictors. In addition, a complementary estimation based on the margin of error approach was performed, assuming a 95% confidence level, a margin of error of 0.05, and maximum variance (*p* = 0.5), which yielded a target sample size of approximately 462 participants, accounting for an anticipated dropout rate. As outlined in an earlier report [13], the rationale underlying sample size considerations has already been presented. The present analytical sample consisted of 418 students after eligibility screening and exclusion of incomplete cases. Considering standard multivariate estimation principles and the consistency of parameter estimates obtained, the available sample was regarded as adequate for the intended structural analyses.

### 2.4. Participants

Recruitment across two consecutive cohorts resulted in 454 enrolled students. After implementation of screening criteria and removal of incomplete cases, the final sample comprised 418 individuals (199 men, 219 women). All retained participants had complete data on primary behavioral measures, and any minor missingness was addressed using the imputation approach outlined in Section 2.7.

The target group included full-time university students aged 18–25 years enrolled in sport- and health-related academic programs and participating in standard in-person instruction. Due to the curricular structure and institutional profile, students in these programs are generally characterized by higher habitual physical activity levels compared with the broader university population. The term “physically active,” where used in the manuscript, refers to this academic context rather than to a predefined quantitative IPAQ threshold used as an inclusion criterion.

Exclusion was applied to individuals involved in institutional competitive sports pathways, those with diagnosed chronic metabolic/psychiatric disorders, night-shift workers, and respondents exhibiting implausible survey patterns.

**Figure 1:**
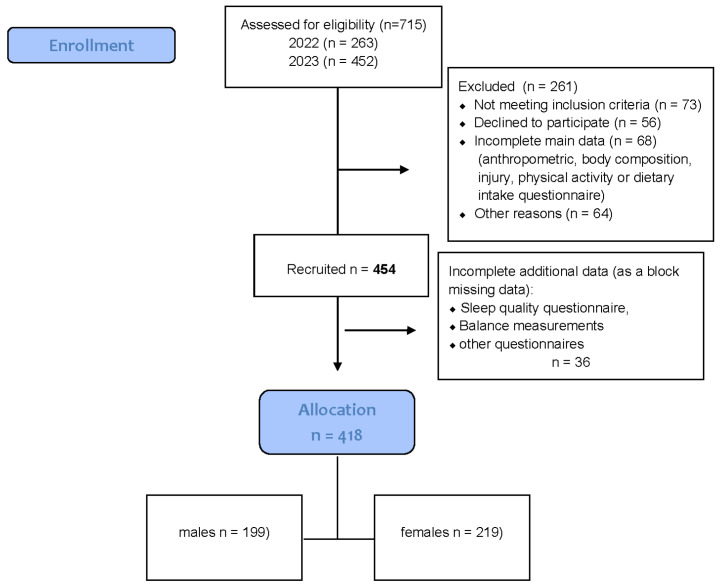
Flow diagram of the progress through the all phases of data collection.

The two cohorts were evaluated for comparability prior to data pooling, with no significant differences observed in age, sex distribution, BMI, dietary variables, or physical activity levels (all *p* > 0.05), supporting their equivalence. Figure 1 presents the flow diagram for participant inclusion in the current analysis.

### 2.5. Anthropometric Measurements

Measurements of body dimensions were obtained at the Biokinetics Research Laboratory, operating within the Central Research Laboratory of the Wroclaw University of Health and Sport Sciences. Height was determined twice using a calibrated GPM anthropometer (GPM Instruments GmBH, Susten, Switzerland), with precision maintained at 0.1 cm. Body mass and body fat mass was measured to the nearest 0.1 kg using an bioimpedance InBody230 device (InBody Co. Ltd., Seoul, Republic of Korea). Measurements were performed under standardized laboratory conditions. Participants were instructed to avoid vigorous physical activity and large meals prior to assessment. Assessments were conducted during scheduled academic hours under consistent procedural conditions, following manufacturer guidelines, to minimize variability related to hydration status and recent physical activity. Fat Mass Index (FMI) was calculated as fat mass (kg) divided by height squared (m^2^).

### 2.6. Questionnaire Measurements

#### 2.6.1. Social-Economic Status—Family Affluence Scale (FAS)

Socioeconomic status was assessed using the Family Affluence Scale III (FAS III), administered electronically via Google Forms (Jaroslaw Domaradzki “testy-PA-SES-INJ_2023” The questionnaire is available from the author upon reasonable request). FAS III is a validated, asset-based indicator of family socioeconomic position, designed as an alternative to traditional measures such as parental income or occupation and shown to be particularly suitable for use in young adult populations [14].

The scale comprises six items reflecting material household resources, including ownership of cars, computers or tablets, dishwashers, number of bathrooms, having a private bedroom, and frequency of family holidays. The FAS III reflects family-level material affluence and does not assess current personal income, employment status, or financial autonomy of participants. In the present study, it was used as an indicator of distal socioeconomic background rather than contemporaneous individual economic conditions. Total FAS III scores were calculated by summing all item responses, yielding a range from 0 to 13 points; higher totals denote more favorable socioeconomic conditions. The Polish version of the instrument was employed [15]. Internal consistency reached α = 0.64, and the score distribution exhibited a modest negative skewness (−0.41). Although Cronbach’s alpha was moderate (α = 0.64), this level is consistent with previously reported values for FAS III in young adult samples. As FAS represents an asset-based composite index rather than a reflective latent construct, internal consistency is not the primary criterion of validity, and the scale is typically analyzed as an observed summary score.

#### 2.6.2. Physical Activity—International Physical Activity Questionnaire (IPAQ)

Self-reported physical activity was collected using the electronically administered Polish IPAQ-LF [16]. Activity data were converted into weekly MET values (MET-min/week) to quantify total movement volume and sedentary exposure. In this study, IPAQ outputs were incorporated as behavioral indicators in the structural model rather than applied for exercise intensity or training-load stratification.

#### 2.6.3. Dietary Intake Questionnaire—Questionnaire Eating Behaviors (QEB)

Dietary intake over the preceding 12 months was assessed using the food-frequency module of the Questionnaire of Eating Behaviors (QEB), applied in its recommended 16-item core version [17,18]. Each item captured consumption frequency of a specific food group using six ordered response categories (never; 1–3 times per month; once per week; several times per week; daily; several times per day). For modeling purposes, individual QEB items were incorporated as behavioral predictors within the structural framework.

Two composite dietary indices were constructed based on the predefined structure of the Questionnaire of Eating Behaviors (QEB). The QEB includes 16 dietary items, of which eight represent pro-healthy food groups and eight represent less healthy, energy-dense or highly processed products. The positive dietary index (DIPOS) comprised eight items reflecting regular consumption of nutritionally favorable foods (wholegrain bread, milk, fermented milk drinks, curd cheese, fish and fish dishes, legumes, fruits, and vegetables). The negative dietary index (DINEG) included items reflecting frequent intake of energy-dense or highly processed foods (fast food, fried foods, cheese including cream cheese, sweets and confectionery, canned meat and fish products, sweetened carbonated beverages, energy drinks, and alcoholic beverages). For each item, consumption frequency was converted into daily equivalents according to the QEB scoring protocol. Each index was calculated as the sum of daily consumption frequencies of the respective eight items, resulting in two continuous composite indicators. Higher values indicated greater adherence to the corresponding dietary pattern. No additional weighting or standardization was applied at the index construction stage. Both indices were subsequently z-standardized for inclusion in the structural equation models. The internal reliability of the QEB has been reported as good to very good (Fleiss’ κ = 0.64–0.84) [17].

### 2.7. Handling and Imputation of Missing Data

Missing data were minimal and concerned a small number of dietary observations. Preliminary diagnostics supported the assumption of missing completely at random (MCAR) [19]. A multiple imputation framework using chained equations (MICE v3.14.0 in R v. 4.3) was applied prior to model estimation [20]. Twenty imputed datasets were generated, and parameter estimates were pooled according to Rubin’s rules. All variables included in the structural model were incorporated into the imputation procedure. Structural equation models were estimated separately within each imputed dataset using the lavaan package (v. 0.6-21) in R, and pooled parameter estimates and standard errors were obtained according to Rubin’s rules.

In the final analytical sample used for the present SEM analyses (n = 418), no missing values were present in the variables included in the structural models; thus, SEM results were effectively based on complete-case data for the analyzed variables.

### 2.8. Statistics

All study variables were summarized using descriptive measures, including arithmetic means, standard deviations, and corresponding 95% confidence intervals. Given that part of the data exhibited non-normal distributions, distributional assumptions were assessed using the Shapiro–Wilk test, and medians were additionally reported to provide a robust description of central tendency. Importantly, deviations from normality were not considered critical for the main analyses, as the primary analytical approach was based on structural equation modeling (SEM) using robust estimation methods. However, for preliminary sex comparisons presented in Table 1, selected QEB-derived variables were subjected to Yeo–Johnson transformation to reduce skewness and improve distributional comparability between males and females. These transformations were applied solely for descriptive and group-comparison purposes and were not used in the structural equation modeling, which relied on z-standardized variables and robust maximum likelihood estimation.

In the present study, the term “lifestyle behaviors” refers specifically to four observed indicators: total physical activity (IPAQ MET-min/week), sedentary time (SITT), positive dietary index (DIPOS), and negative dietary index (DINEG).

As a preliminary analysis, associations between the independent variable (family affluence, FAS) and behavioral mediators (physical activity, sedentary time, positive and negative dietary indices) were examined using Pearson’s, Spearman’s, and Kendall’s tau correlation coefficients. These analyses were conducted to explore the strength and direction of associations and to inform the specification of the SEM model.

The main analysis was performed using structural equation modeling (SEM). The model specified direct paths from family affluence to behavioral variables and from each behavioral factor to fat mass index (FMI), as well as a direct path from family affluence to FMI. The primary objective of the model was to compare the relative magnitude of direct associations between family affluence and concurrent lifestyle indicators within a single multivariable framework. Although indirect pathways were conceptually plausible, preliminary correlations between family affluence and behavioral variables were weak. To address the possibility of mediation, additional sensitivity analyses were conducted including formal indirect pathways (FAS → PA → FMI; FAS → dietary indices → FMI). These indirect effects were small and statistically non-significant in both sexes, and inclusion of mediation chains did not materially alter parameter estimates or model fit. Models were estimated separately for males and females. Formal multi-group invariance testing and χ^2^ difference comparisons of path coefficients across sexes were not conducted, as the primary objective was to describe sex-specific association patterns rather than to test equality constraints between groups. Standardized path coefficients (β), standard errors (SE), 95% confidence intervals, and coefficients of determination (R^2^) were reported for the outcome variable. To account for deviations from normality, robust maximum likelihood estimation was applied. Model fit indices (χ^2^, *df*, *p*, CFI/TLI, RMSEA with CI, SRMR) were reported. Because the primary direct-effects models were just-identified (*df* = 0) due to freely estimated covariances among predictors, fit indices were additionally evaluated in a sensitivity model including plausible mediation chains (FAS → PA → FMI; FAS → dietary indices → FMI). Formal multi-group invariance testing between sexes was not performed; therefore, differences in path coefficients between males and females are interpreted descriptively rather than as statistically tested group effects.

All analyses were conducted in Statistica 14.0 (TIBCO Software Inc., Palo Alto, CA, USA) and RStudio (2025.09). Structural equation modeling was conducted in R using the lavaan package (v. 0.6-21). Models were estimated using robust maximum likelihood (MLR) to reduce sensitivity to non-normality. Sex-specific models were fit separately for males and females within each imputed dataset, and pooled estimates were obtained using Rubin’s rules. Statistical significance was set at *p* < 0.05.

### 2.9. AI Transparency Statement

The authors used generative AI tools (including ChatGPT: OpenAI, GPT-4.1, 2025) for language refinement, structural editing, and assistance with literature organization. AI tools were not involved in study design, data collection, statistical analysis, or interpretation of results. All outputs were critically reviewed and verified by the authors, who take full responsibility for the scientific integrity and final content of the manuscript.

## 3. Results

### 3.1. General Characteristics of the Study Sample

Descriptive statistics and comparisons between males and females in anthropometric measurements, physical activity (IPAQ) and dietary behaviors (16 items from QEB) have been presented previously in Domaradzki 2025 [21]. Sex-stratified comparisons revealed that men were characterized by greater height, body mass, BMI (all *p* < 0.001), and higher overall physical activity levels (*p* < 0.001). Conversely, women demonstrated higher frequency of consumption of wholegrain products, milk and fermented dairy items, curd cheese, legumes, fruits, and vegetables (all *p* ≤ 0.02), indicating distinct dietary profiles between sexes. Males more often reported consumption of fast food, fried meals, yellow cheese, canned meals, sweetened beverages, energy drinks and alcoholic drinks (all *p* < 0.001). No significant sex differences were found for fish or sweets (*p* > 0.05).

Table 1 presents all variables included in the SEM analysis. Apart from the results related to physical activity (PA) and fat mass index (FMI) reported above, no significant sex differences were observed in family affluence (FAS) or sedentary time (SITT) (*p* > 0.05). Females scored higher on the positive dietary index (DIPOS), whereas males scored higher on the negative dietary index (DINEG) (both *p* < 0.001).

### 3.2. Preliminary Correlation Analysis

Table 2 presents the results of preliminary correlation analyses conducted to inform the specification of the SEM model. Pearson’s, Spearman’s, and Kendall’s tau coefficients were calculated separately for males and females to examine associations among family affluence and behavioral variables.

Overall, correlations between family affluence (FAS) and behavioral indicators were weak and largely non-significant in both sexes, with coefficients close to zero. Associations between physical activity (PA) and sedentary time (SITT) were small in magnitude in both males and females.

The most consistent relationships were observed between dietary indices, where moderate negative correlations between positive (DIPOS) and negative (DINEG) dietary patterns were evident across sexes. In females, moderate associations were also observed between sedentary time and dietary variables; however, these remained domain-specific rather than cross-domain effects.

Taken together, the generally weak cross-domain correlations and the concentration of moderate associations primarily within dietary constructs indicate a limited degree of interdependence among behavioral predictors. Consequently, mediation pathways among behavioral variables were not specified in the SEM models, which focused on their independent direct associations with fat mass index (FMI).

### 3.3. Structural Equation Modeling

**Figure 2:**
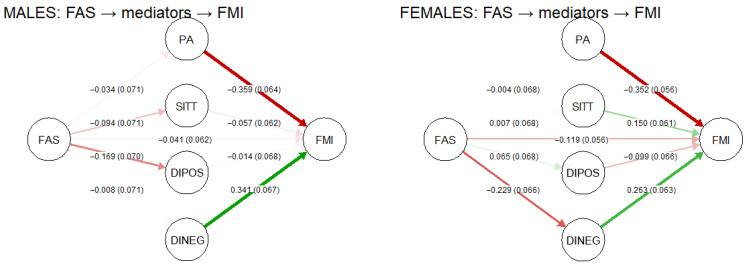
Path diagram illustrating associations between family affluence (FAS), lifestyle indicators, and fat mass index (FMI). Standardized path coefficients (β) with standard errors (SE) are shown for direct effects from FAS to physical activity (PA), sedentary time (SITT), positive dietary index (DIPOS), and negative dietary index (DINEG), as well as for direct effects of these mediators and FAS on FMI. Models were estimated separately for males and females.

In males, fat mass index was independently associated with physical activity (β = −0.36, *p* < 0.001) and negative dietary index (β = 0.34, *p* < 0.001), jointly explaining 30% of FMI variance (Table 3). Sedentary time, positive dietary behaviors, and family affluence showed no direct effects after mutual adjustment. Physical activity was moderately correlated with both positive (r = 0.28) and negative (r = −0.25) dietary indices, indicating clustering of health-related behaviors. The standardized path coefficients for males are illustrated in Figure 2.

In females, fat mass index was independently associated with physical activity (β = −0.35, *p* < 0.001), sedentary time (β = 0.15, *p* = 0.022), and negative dietary index (β = 0.26, *p* = 0.001), jointly explaining 37% of FMI variance (Table 3). Positive dietary behaviors and family affluence showed no direct effects. Sedentary time was strongly correlated with both dietary indices, indicating a central role of inactivity in the clustering of unhealthy behaviors among females.

Although coefficients were estimated separately for males and females, no formal χ^2^ difference test or multi-group invariance analysis was conducted; thus, apparent differences between sexes should be interpreted cautiously as descriptive comparisons.

Model fit. The primary sex-specific SEM was specified as a direct-effects path model with freely estimated covariances among predictors, resulting in a just-identified structure (*df* = 0) in both males and females. Consequently, global fit indices are perfect by definition (χ^2^ = 0, CFI/TLI = 1.00, RMSEA = 0, SRMR = 0) and do not provide an informative test of model adequacy. Therefore, model fit was additionally evaluated using a sensitivity model that included plausible indirect paths (FAS → PA → FMI; FAS → dietary indices → FMI), yielding an overidentified structure (*df* = 1) with acceptable-to-excellent fit in both sexes (males: χ^2^_scaled(1) = 2.213, *p* = 0.137; CFI_robust = 0.993; RMSEA_robust = 0.069; SRMR = 0.019; females: χ^2^_scaled(1) = 0.014, *p* = 0.907; CFI_robust = 1.000; RMSEA_robust = 0.000; SRMR = 0.002). Indirect effects were small and non-significant in both sexes (all *p* ≥ 0.071), supporting the interpretation that associations with FMI were primarily driven by direct behavioral paths.

To evaluate potential multicollinearity between dietary indices and other predictors, variance inflation factors (VIF) were calculated using linear regression models. All VIF values were low (range: 1.05–1.46), indicating no evidence of problematic collinearity.

## 4. Discussion

The present study demonstrates that current lifestyle indicators (physical activity, sedentary time, and dietary indices)exert a substantially stronger and more consistent influence on fat mass index (FMI) than family affluence (FAS) in young adults. Across both sexes, physical activity emerged as the strongest protective factor, showing robust inverse associations with FMI, while the negative dietary index represented the most influential adverse determinant. In contrast, positive dietary behaviors and family affluence showed weak or non-significant direct associations with FMI, particularly in males. Although a modest direct effect of FAS on FMI was observed in females, its magnitude was small compared with the effects of physical activity and unhealthy dietary behaviors. These findings suggest that, in this population, proximal, modifiable behaviors were more strongly associated with FMI than distal family background factors in shaping adiposity outcomes.

The present findings raise the question of whether family-related habits are still expected to manifest during early adulthood, or whether they are progressively replaced by new behavioral patterns shaped by the academic and social environment. Conceptually, family affluence reflects long-term household conditions and early-life exposures, whereas the analyzed lifestyle indicators (physical activity, sedentary time, and dietary indices) capture current, situational lifestyle patterns. Young adults health behaviors are influenced by socioeconomic factors, but these influences vary, suggesting that family-related habits may not strongly persist into adulthood [22]. However, evidence from studies suggest that socioeconomic status (SES) measures are correlated with unhealthy behaviors among young adults [23].

Participants in this study were assessed during an academic semester, a period characterized by increased autonomy, altered daily routines, and reduced parental control. Under these circumstances, the influence of family-related socioeconomic conditions may be reduced, whereas individual behavioral choices may play a more prominent role; however, such mechanisms were not directly measured in the present study and should therefore be interpreted cautiously. Many studies suggest that individual behavioral choices may become more influential of body composition during the academic semester, with reduced parental influence and increased personal autonomy [24,25,26]. Annesi et al. 2020 [27] specifically highlighted semester-related increases in anxiety-related eating and decreases in self-regulation, further supporting the notion that individual behavioral choices may contribute to variation in body composition during this transitional period. The slightly stronger association between FAS and FMI observed among females may reflect greater persistence of family-related health behaviors or gender-specific sensitivity to socioeconomic contexts; however, even in females, the effect of FAS remained smaller than the associations observed for physical activity and negative dietary behaviors [28,29].

Correlation analyses revealed weak or non-significant associations between family affluence (FAS) and health behaviors, including physical activity (PA), sedentary time (SITT), positive dietary index (DIPOS), and negative dietary index (DINEG). The only consistent association was the expected inverse relationship between DIPOS and DINEG, indicating partial opposition between healthy and unhealthy dietary patterns. Overall, the correlation structure did not support strong interdependencies among mediators, thereby limiting the potential for indirect effects. Comparable findings have been reported in population-based studies of early adulthood, where physical activity and dietary behaviors often function as relatively independent lifestyle domains. Even under clear socioeconomic stratification, correlations between PA and diet tend to remain weak and explain limited shared variance in adiposity outcomes [30]. In contrast, some studies have demonstrated stronger associations between socioeconomic status and dietary behaviors or sedentary time, particularly in populations characterized by pronounced social inequalities. In such contexts, SES may structure clusters of behaviors combining low physical activity, poor diet quality, and higher sedentary exposure [31]. Weak correlations between mediators may reflect the increasing autonomy of lifestyle choices in early adulthood. Physical activity and dietary behaviors are shaped by distinct social contexts (school, peers, leisure environments) and may no longer be directly constrained by family socioeconomic background, resulting in fragmentation rather than clustering of health behaviors.

Path analysis revealed significant direct associations between lifestyle indicators and FMI across sexes: physical activity showed an inverse relationship, while negative dietary behaviors exhibited a positive association. Effects of positive dietary behaviors and sedentary time were weak or non-significant. Family affluence did not exert significant indirect effects through PA, SITT, or dietary indices. Only in females was a small but statistically significant direct effect of FAS on FMI observed. Evidence from structural equation modeling in adult populations suggests that health behaviors—particularly diet and sedentariness—can mediate the association between socioeconomic position and adiposity-related outcomes [28,29]. In a large population-based study of 3995 Finnish males and females, dietary behaviors and sedentary time significantly mediated the relationship between socioeconomic position and BMI, indicating that socioeconomic disparities in adiposity were largely explained by behavioral pathways rather than direct effects [32]. The discrepancy between such findings and the absence of mediation in the present study may reflect both developmental and methodological differences across studies. Additionally, the relatively homogeneous socioeconomic profile of the sample may have limited variability in family affluence (range restriction), potentially attenuating detectable indirect effects. Restricted variance in FAS may reduce statistical power to detect mediation pathways, even if such mechanisms exist in more socioeconomically heterogeneous populations.

From a developmental perspective, early adulthood represents a transitional stage characterized by increasing behavioral autonomy and weaker anchoring of daily habits in family socioeconomic background. In contrast, adult samples may capture more stable, accumulated socioeconomic gradients that structure long-term lifestyle patterns. Methodologically, differences in analytical strategies—such as regression-based mediation models versus simultaneous SEM frameworks estimating direct and indirect paths concurrently—may also contribute to divergent mediation patterns reported across studies.

At the same time, young adults-focused studies consistently demonstrate that physical activity and unhealthy dietary behaviors exert strong direct effects on adiposity-related outcomes, often remaining dominant even when socioeconomic indicators are included in multivariable or SEM frameworks [33]. Studies focusing on young adults suggest that meal irregularity combined with energy-dense food consumption contributes to greater fat accumulation and a heightened propensity toward obesity [25,34]. In contrast to the strong behavioral mediation observed in adult populations, the present study did not identify significant indirect pathways linking family affluence to FMI through physical activity, sedentary behavior, or dietary indices. This divergence is consistent with evidence from young adults samples indicating that socioeconomic indicators may sometimes explain more variance in body composition than measured dietary intake or activity behaviors [35,36].

Physical activity showed the strongest protective effect on FMI, whereas negative dietary behaviors exerted the strongest adverse effect. This hierarchy of effects is consistent with prior research identifying physical activity and unhealthy dietary behaviors as the primary predictors of young adults adiposity, even across different socioeconomic strata [33,37,38]. Where structural socioeconomic barriers limit availability of healthy dietary options and physical activity resources, SES often assumes a stronger role in shaping adiposity-related outcomes [31]. Recent scoping evidence in university populations further supports the relevance of examining physical activity and dietary behaviors jointly rather than in isolation. Vasco et al. (2025) [39] emphasized that combined lifestyle patterns are associated not only with body composition, but also with psychological well-being, cognitive performance, and quality of life, with partially sex-specific pathways. Although the present study focused on adiposity outcomes, the observed clustering of behavioral indicators and sex differences in sedentary behavior are conceptually consistent with this broader integrative framework [39].

The discrepancy between the strong mediation observed in adult populations and the absence of indirect effects in the present young adults sample may therefore reflect developmental context. While socioeconomic position in adulthood strongly structures daily routines and long-term habits, early adulthood is characterized by greater behavioral variability and weaker anchoring of lifestyle behaviors in family affluence [32]. Stronger effects of physical activity and unhealthy diet may reflect their direct physiological relevance to energy balance and fat accumulation, whereas socioeconomic effects are more diffuse, context-dependent, and moderated by developmental stage. Taken together, the findings indicate that lifestyle indicators showed stronger direct associations with FMI in the studied population, whereas the role of family affluence was not statistically significant within the tested cross-sectional model, particularly in early adulthood. These findings highlight the importance of behavior-focused interventions targeting physical activity and dietary quality in physically active university students and comparable academic environments. Given that the sample consisted of students enrolled in sport- and health-related programs, baseline levels of physical activity may have been relatively high and less variable than in the general university population. This sample profile may reduce variability in both behavioral and socioeconomic indicators and should be considered when interpreting the absence of a detectable direct association between family affluence and FMI.

Several limitations should be acknowledged. First, information on living arrangements was not available, precluding differentiation between students residing in the parental household and those living in dormitories or rented accommodations. This distinction may be particularly relevant when interpreting the attenuated role of family affluence, as the degree of ongoing parental influence likely varies across living contexts. Second, the cross-sectional design constrains interpretation to associations rather than causal relationships and does not permit examination of longitudinal transitions from family-shaped to autonomous health behaviors. In addition, reliance on self-reported measures of physical activity, sedentary time, and diet may entail measurement error related to recall limitations and social desirability tendencies. Such self-report instruments (e.g., IPAQ and food-frequency–based dietary indices) are also prone to systematic misreporting, including overestimation of physical activity and underreporting of unhealthy food intake, which may differ by sex and body composition status. This potential bias should be considered when interpreting the magnitude of observed associations. Third, although bioimpedance analysis is suitable for field-based research, it may be influenced by hydration status and recent physical activity. Furthermore, the models did not include several potentially relevant covariates, such as smoking status, alcohol consumption, detailed training load, total energy intake, or field of study specialization. In particular, the absence of direct assessment of total energy consumption limits the ability to disentangle qualitative dietary patterns from overall caloric balance, which is directly related to adiposity. These factors may influence fat mass index and could correlate with both socioeconomic background and behavioral patterns. Their absence raises the possibility of residual confounding, which may partly account for the explained variance (R^2^ ≈ 30–37%) and may have influenced the magnitude or detectability of associations, including the absence of a statistically significant direct effect of family affluence on FMI. An additional limitation concerns the institutional profile of the sample. Participants were recruited from the Wroclaw University of Sport and Health Sciences, and the target group consisted of students enrolled in sport- and health-related programs. As a result, the sample may present relatively homogeneous and elevated levels of physical activity compared with the general university population. This recruitment context may introduce selection bias and restrict variability in key predictors, potentially attenuating observable associations. Therefore, caution is warranted when generalizing the findings to broader and more heterogeneous university populations. Moreover, the study did not assess current personal income, employment status, or financial autonomy, which may be particularly relevant in early adulthood when economic independence gradually increases. Therefore, the absence of a direct association between FAS and FMI should be interpreted in the context of family-based rather than individual-level socioeconomic measurement. Future studies incorporating longitudinal designs, objective behavioral measures, and detailed contextual data on living arrangements and food environments are warranted.

## 5. Conclusions

In conclusion, this study indicates that physical activity and negative dietary behaviors were the strongest direct correlates of fat mass index in young adults within the present cross-sectional framework. In contrast, family affluence did not demonstrate a meaningful direct association with FMI in the tested models, and behavioral mediation pathways were not supported under the specified structure. These findings suggest that, during the university period, current lifestyle indicators may be more closely associated with adiposity than distal socioeconomic background. Accordingly, intervention strategies targeting physical activity and dietary quality may be particularly relevant in this population, namely physically active university students enrolled in sport- and health-related programs, and should be generalized to broader student populations with caution.

## Figures and Tables

**Table 1:** Anthropometric parameters and questionnaire-derived scores (PSQI, QEB, IPAQ) are presented separately for males and females. QEB variables were normalized using the Yeo–Johnson power transformation.

	Sex = Males, N = 199					Sex = Females, N = 219						
Variables	Me	Mean	95% CI		SD	Me	Mean	95% CI		SD	t	*p*
			Lower	Upper				Lower	Upper			
FAS [scores]	9.0	8.6	8.4	8.8	1.4	9.0	9.0	8.8	8.6	9.0	−1.48	0.141
IPAQ [MET/minutes/week]	3364.6	3608.2	3418.5	3797.9	1356.9	2849.0	3019.8	2886.5	3153.1	1000.9	**5.08**	**0.000**
FMI [kg/m^2^]	3.8	3.9	3.7	4.1	1.4	4.8	5.1	4.9	5.3	1.8	**−7.33**	**0.000**
SITT [minutes/week]	1498.0	1604.4	1509.5	1699.3	679.0	1386.0	1592.4	1484.4	1700.4	810.8	0.16	0.870
DIPOS [scores]	2.7	2.9	2.8	3.1	1.3	4.3	4.3	4.1	4.5	1.5	**−9.34**	**0.000**
DINEG [scores]	2.9	2.9	2.7	3.0	1.3	1.5	1.6	1.5	1.7	0.9	**12.32**	**0.000**

Footnote: Me—median; FAS—Family Affluence Scale; IPAQ—International Physical Activity Questionnaire; FMI—fat mass index; SITT—sitting time; DIPOS—positive dietary behaviors; DINEG—negative dietary behaviors. Statistically significant differences are in bold font.

**Table 2:** Pearson’s, Spearman’s, and Kendall’s tau correlation coefficients among family affluence (FAS), physical activity (PA), sedentary time (SITT), and dietary indices (DIPOS and DINEG), stratified by sex.

Males										
Correlation	FAS-PA	FAS-SITT	FAS-DIPOS	FAS-DINEG	PA-SITT	PA-DIPOS	PA-DINEG	SITT-DIPOS	SITT-DINEG	DIPOS-DINEG
Pearson	−0.03	−0.09	−0.17	−0.01	−0.18	0.28	−0.25	−0.11	0.18	−0.41
Spearman	−0.02	−0.14	**−0.16**	−0.01	**−0.25**	**0.32**	**−0.30**	**−0.23**	**0.21**	**−0.40**
Kendall	−0.01	−0.10	**−0.12**	−0.01	**−0.18**	**0.23**	**−0.22**	**−0.16**	**0.14**	**−0.28**
**Females**										
Pearson	0.01	0.01	0.07	**−0.23**	**−0.15**	**0.20**	**−0.16**	**−0.44**	**0.29**	**−0.45**
Spearman	−0.08	0.03	0.06	**−0.16**	−0.10	**0.14**	−0.11	**−0.50**	**0.38**	**−0.55**
Kendall	−0.06	0.03	0.04	**−0.11**	−0.07	**0.09**	−0.07	**−0.35**	**0.26**	**−0.39**

Footnote: FAS—Family Affluence Scale; SITT—sitting time; DIPOS—positive dietary behaviors; DINEG—negative dietary behaviors. Statistically significant correlations are in bold font.

**Table 3:** Standardized path coefficients from sex-specific structural equation models examining associations of physical activity (PA), sedentary time (SITT), dietary indices (DINEG, DIPOS), and family affluence (FAS) with fat mass index (FMI). Models were estimated separately for males and females using z-standardized variables.

Path (→FMI)	β (SE)	95% CI	*p*	β (SE)	95% CI	*p*
		**Males (n = 199)**			**Females (n = 219)**	
PA → FMI	−0.359 (0.053)	[−0.464; −0.255]	<0.001	−0.352 (0.052)	[−0.453; −0.250]	**<0.001**
SITT → FMI	−0.057 (0.049)	[−0.153; 0.039]	0.248	0.150 (0.065)	[0.022; 0.278]	**0.022**
DINEG → FMI	0.341 (0.069)	[0.205; 0.477]	<0.001	0.263 (0.082)	[0.102; 0.424]	**0.001**
DIPOS → FMI	−0.014 (0.072)	[−0.155; 0.128]	0.848	−0.099 (0.069)	[−0.235; 0.036]	0.151
FAS → FMI	−0.041 (0.057)	[−0.153; 0.070]	0.469	−0.119 (0.082)	[−0.279; 0.041]	0.146
R^2^ (FMI)	0.301			0.373		

Footnote: Statistically significant effects are in bold font.

## Data Availability

The data presented in this study are available on request from the author.
